# Multivariate analysis of teams’ physical performances in official football matches in LaLiga 2023–24

**DOI:** 10.5114/biolsport.2025.146784

**Published:** 2025-02-04

**Authors:** Julen Castellano, Sergi Bellmunt, Ricardo Resta, Roberto Lopez del Campo, David Casamichana

**Affiliations:** 1 GIKAFIT research group, University of the Basque Country (UPV/EHU), Vitoria-Gasteiz, Spain; 2 HUDL Company; 3 Department of competitions and Mediacoach, LaLiga, Madrid, Spain; 4 Real Sociedad Institute, Real Sociedad de Fútbol S.A.D., Donostia-San Sebastián, Spain

**Keywords:** Soccer, Performance, Time-motion analysis, Team sport, Monitoring

## Abstract

The main aims of this study were to describe the physical performance of the teams during official matches using a multivariate approach, considering their rivals and the final competition standings. The study analysed the professional teams that competed in the first division of Spanish football during the 2023–2024 season. A total of 756 physical performances of teams were analysed across 378 matches. Data for nine external match load variables were collected using the TRACAB optical tracking system: total distance and distance covered at high speeds (> 21, > 24 and > 28 km·h^-1^), total acceleration load, and the frequency of accelerations/decelerations (> 3 and > 4 m·s^2^). Principal component analysis (PCA) and clustering analysis were used to reduce multidimensionality and facilitate grouping. 1) PCA grouped the external load variables into three components: intensity (characterized by a high number of accelerations and decelerations), velocity (characterized by high values in high-speed distance), and volume (characterized by a high total distance). 2) Teams’ physical performances were primarily grouped into four clusters: cluster 1 (high intensity), cluster 2 (highest values across all physical variables), cluster 3 (lowest values across all physical variables), and cluster 4 (high velocity). 3) No significant differences were found in the distribution of physical performances within each cluster based on the teams’ final rankings. 4) Teams’ physical performances showed a tendency to play most of their matches against opponents from the same cluster. The clustering analysis revealed differences in physical demands across teams during the season, which can guide training and match preparation. Teams can use this knowledge to improve injury prevention and recovery management by aligning physical preparation with match external loads.

## INTRODUCTION

Analysis of physical demands in competitive matches in elite football has been one of the most studied topics in recent years [1]. Access to technology in professional clubs has facilitated the availability of information on players’ physical performance [2]. The most common variables are the locomotor metrics accumulated by teams during competition, such as total distance (TD), high-speed running distance, and very high-speed running distance [3, 4].

As the team is usually considered as a unit, approaching the analysis of physical performance from the cumulative physical performance of the 11 players on the field that make up the team, as has been done recently using the locomotor dimension [5], makes perfect sense. However, studies providing data on the accumulated load in the neuromuscular dimension for the team still need to be included [1]. Given that some physical variables show a low correlation with each other, e.g., high speed versus changes of velocity [6], a multivariate analysis of representative variables from both dimensions could provide valuable insights into the type of physical demands present in official matches throughout an entire season.

In the information age, with so much data available, analytical approaches to address multidimensionality seem necessary. To reduce multidimensionality and facilitate grouping, principal component (PCA) and cluster analysis are appropriate. PCA has been applied in the world of football in different contexts. Specifically, it has been applied to the analysis of external load variables of training tasks [7] and training sessions [8] or styles of play in official matches [9], although usually in the context of a single team, and therefore it is not very generalizable, unless many case studies are added. The advantage of implementing clustering is that it allows the identification of groups according to multiple variables, as in the case of Australian soccer, where typologies of players were identified from forty-six variables [10] or to determine the number of groups based on the position in professional rugby players based on physical, technical, or physical-technical variables [11]. In the specific case of the physical demand of an official match, it could allow us to ascertain the type of physical demand that a team presents more frequently or the one it has had in a specific match, simplifying the interpretation of the results. However, to date it has yet to be applied in the study of the physical demands of all the matches played by all the teams in a competition.

Furthermore, the relationship between physical match demands and performance in an entire season has been studied by different studies previously [12, 13]. In most cases, no clear relationship has been found between external match demands and the position occupied by the teams in the final classification [14], which indicates that other technical-tactical elements, or their combination, are the ones that have the most influence on the performance. However, to the authors’ knowledge, in no case has a multivariate analysis and grouping through clusters of the physical demands of the teams during official matches been used.

It has recently been reported that the physical response of a team in a competitive match, in addition to the context variables [15], depends mainly on the physical response of the opposing team [16]. In the attempt to describe this physical response, it seems interesting to know to what extent the teams of a particular league can be aware of the physical demand scenarios that can be expected during the competition.

Taking into account the potentially novel aspects of the above, the objective of this study was to describe the physical performance of the teams during the official matches using a multivariate approach and, complementarily, to evaluate the association between the physical performance of the teams with their rivals and the final classification in the competition. The results would allow us to know how teams deploy their physical response and, at the same time, to know the number and type of match physical demand that each team accumulates during the season.

## MATERIALS AND METHODS

### Design

This was a descriptive observational study of the physical performance of the teams belonging to the first division of Spanish soccer (LaLiga) during the 2023–2024 season. The competition began on August 11, 2023 and ended on May 26, 2024, with a duration of nine months.

### Participants

The sample included 20 professional male football teams that competed in the LaLiga, including the analysis of 756 observations of team performances during 378 official matches (~99% of the total possible team performances). Each team’s collective total physical performance during each official match was registered. Four team performances that had incomplete information were discarded from the analysis. The dataset utilized in this investigation was provided by LaLiga, which authorised the analysis of pertinent variables and the dissemination of results for scientific purposes. In strict adherence to the ethical guidelines outlined by LaLiga, the present investigation does not contain any information that could lead to the identification of individual football players. Furthermore, all procedures and protocols employed in this study were conducted according to the principles outlined in the Declaration of Helsinki.

### Independent variables

The independent variables or factors were the teams and the final ranking. This second factor was grouped into performances of teams that managed to qualify for Europe, those that remained in the category and those that were relegated.

### Measures

To evaluate the external load of teams on each match day accumulated by teams, nine dependent variables were used: 1) total distance (TD, m): the number of metres that the team covered during the match; 2) high-speed running (TD21, m): the number of metres that the team covered at > 21 km · h^−1^ during the match; 3) sprint (DT24, m): the number of metres that the team covered at > 24 km · h^−1^ during the match; 4) sprint at high velocity (TD28, m): the number of metres that the team covered at > 28 km · h^−1^ during the match; 5) acceleration load (aLoad, au): it refers to the total amount of acceleration accumulated over a specific period of time; 6) accelerations (ACC3, n): the number of accelerations that the team makes at > 3 m · s^−2^ during the match; 7) decelerations (DEC3, n): the number of decelerations that the team makes at < -3 m · s^−2^ during the match; 8) accelerations at high intensity (ACC4, n): the number of accelerations that the team makes at > 4 m · s^−2^ during the match; and, 9) decelerations at high intensity (DEC4, n): the number of decelerations that the team makes at < -4 m · s^−2^ during the match. These types of variables are usually chosen to describe the physical performance of teams in competition, especially in the league studied in the current study [5, 14, 16, 19].

### Procedures

External load data during the official matches were captured using the TRACAB (ChyronHego, New York, USA) multicamera computerized optical tracking system, which has a sampling frequency of 25 Hz, and processed using the Mediacoach software (LaLiga, Madrid, Spain), which is a valid and reliable system to analyse football performance [17]. The data of this tracking system have been used in previous studies [18, 19].

### Statistical analysis

Three types of analysis were implemented. First, a descriptive analysis was conducted. Cohen’s *d* was calculated for pairwise comparisons among clusters. The following classifications to measure the magnitude of Cohen’s *d* was used (Hopkins et al., 2009): trivial (*d* < 0.2), small (0.2 < *d* < 0.6), moderate (0.6 < *d* < 1.2), large (1.2 < *d* < 2.0) and very large (*d* > 2.0).

Prior to PCA, the external match load variable datasets were examined for suitability for PCA using correlation matrix, Bartlett’s test of sphericity and Kaiser-Meyer-Olkin (KMO) statistics. Bartlett’s test of sphericity was significant (*p* < 0.001) and all variables possessed a Kaiser-Meyer-Olkin global value above 0.73, suggesting that the data were suitable for PCA [8]. Following this, PCA was conducted with data first mean-centred and standardised to unit variance, providing an *M* × *N* matrix (*X*). The covariance matrix of *X* was then computed as X^T^X. The eigenvalues and eigenvectors were determined from the covariance matrix via eigen decomposition. The original mean-centred and standardised data were then projected into the eigenspace (data multiplied by eigenvectors) of the covariance, providing a matrix of PCs often referred to as PC ‘scores’. PCs with eigenvalues > 1 were used as a threshold to determine PCs that were meaningful [20]. Only variables with a loading greater than 0.7 were selected.

Finally, a cluster analysis was performed to group similar physical performance profiles, and the physical demands of the teams during official matches were distributed within these groups. A K-means cluster analysis was conducted using the Euclidean distance metric. Prior to this, the data were standardized. The optimal number of clusters (k) was determined using the elbow method [21], indicating that k = 4 is the optimal inflection point, suggesting that four clusters provide an adequate structure for the data, capturing the main variability without overfitting the model.

Finally, the data were analysed using the chi-square test (χ^2^) of association to evaluate the relationship between [teams] and [cluster], [teams ranking] and [cluster], and [teams cluster] and [opponent cluster]. This test was used due to the categorical nature of the variables, allowing us to determine whether there was a significant association between them. The level of significance was set at p < 0.05. The statistical analysis was conducted using the software Jamovi 2.4.6 for Windows (The jamovi project, 2023).

### RESULTS

Table 1 shows the average values of the external load variables over the season analysed.

**TABLE 1:** Average values, standard deviation (± sd), minimum, maximum and percentiles of the external load variables of the teams in match.

External load variables	mean	sd	minimum	maximum	Percentiles		

					25^th^	50^th^	75^th^
TD (m)	112.614	4.545	92.092	124.929	109.706	112.591	115.604
TD21 (m)	8.778	1.113	5.468	12.129	8.025	8.731	9.550
TD24 (m)	4.052	634	2.445	6.064	3.594	4.006	4.494
TD28 (m)	1.078	263	431	1.920	884	1.051	1.247
aLoad (au)	33.132	1.492	27.337	37.414	32.124	33.226	34.187
ACC3 (n)	704	71.5	503	934	656	704	751
DEC3 (n)	796	77.3	587	996	739	795	848
ACC4 (n)	166	25.5	102	254	148	165	181
DEC4 (n)	269	32.1	187	376	248	268	291

**Note**: TD is total distance, TD21 is distance covered at > 21 km · h^−1^, TD24 is distance covered at > 24 km · h^−1^, TD28 is distance covered at > 28 km · h^−1^, aLoad is acceleration load, ACC3 is number of accelerations at > 3 m · s^−2^, DEC3 is number of decelerations at < -3 m · s^−2^, ACC4 is number of accelerations at > 4 m · s^−2^, DEC4 is number of decelerations at < -4 m · s^−2^, and au is arbitrary units.

Table 2 details the PCA results for the external match load variables. The three PCs accumulated different variances 53.6%, 21.3% and 11.5% for PC1, PC2 and PC3, respectively. The three components accounted for 86.4% of the variance explained. It can be concluded that the first of the components are represented by the variables most associated with accelerations and decelerations (ACC3, DEC3, ACC4, and DEC4), PC2 is represented by the variables associated with high-speed movement (TD21) and sprinting (TD24), with the most significant weight attributed to sprint at high velocity (TD28), and PC3 is represented by the variables most associated with the volume (TD and aLoad).

**TABLE 2:** Principal component analysis results for the external load variables of the teams in match showing eigenvalues, percentage of variance explained, cumulative variance explained, and component loadings for the three principal components.

	Component			Component			

	PC1	PC2	PC3		PC1	PC2	PC3
Eigenvalue	4.83	1.91	1.03	DEC4 (n)	0.773		
% of total variance explained (VE)	53.6	21.3	11.5	TD24 (m)		0.956	
Cumulative % of total VE	53.6	74.9	86.4	TD28 (m)		0.907	
External load variables				TD21 (m)		0.854	
ACC3 (n)	0.913			TD (m)			0.923		
ACC4 (n)	0.875			aLoad (au)			0.709
DEC3 (n)	0.793						

**Note**. PC is principal component, TD is total distance, TD21 is distance covered at > 21 km · h^−1^, TD24 is distance covered at > 24 km · h^−1^, TD28 is distance covered at > 28 km · h^−1^, aLoad is acceleration load, ACC3 is number of accelerations at > 3 m · s^−2^, DEC3 is number of decelerations at < -3 m · s^−2^, ACC4 is number of accelerations at > 4 m · s^−2^, DEC4 is number of decelerations at < -4 m · s^−2^, and au is arbitrary unit.

Four physical performance profiles were distinguished once the cluster analysis was performed, providing a moderate data structure and capturing the main variability without overfitting the model (silhouette score = 0.4). Table 3 shows the average values of each external match load variable according to the cluster to which they belong. As can be seen, cluster 1 is represented by moderate TD, low demand on high-speed range velocity variables (TD21, TD24 and TD28) and high demand on aLoad and accelerations and decelerations. Cluster 2 represents the highest values in all external match load variables, and cluster 3 has the lowest values. Finally, cluster 4, contrary to cluster 1, is represented by a high demand in variables of high-speed range (TD21, TD24, and TD28) and low demand for accelerations and decelerations.

**TABLE 3:** Average values and standard deviation (sd) of the external load variables of the teams in match for each of the four clusters.

External load variables	Clustering				

	1	2	3	4	ES (range)
TD (m)	112,688 (3,594)	**116,467 (4,018)**	108,722 (4,483)	112,516 (3,773)	2 > 1 = 4 > 3 (0.95/1.95)
TD21 (m)	8,234 (656)	**10,003 (766)**	7,757 (749)	**9,450 (658)**	2 > 4 > 1 > 3 (0.68/3.19)
TD24 (m)	3,664 (339)	**4,657 (477)**	3,550 (414)	**4,532 (381)**	2 > 4 > 1 > 3 (0.29/2.78)
TD28 (m)	922 (166)	**1,261 (246)**	929 (184)	**1,287 (195)**	2 = 4 > 1 = 3 (1.71/1.87)
aLoad (au)	**33,683 (925)**	**34,632 (965)**	31,424 (1,140)	32,716 (998)	2 > 1 > 4 > 3 (0.94/3.16)
ACC3 (n)	**733 (46.9)**	**778 (51.4)**	626 (47.8)	682 (39.9)	2 > 1 > 4 > 3 (0.96/3.29)
DEC3 (n)	**825 (48.2)**	**881 (52.1)**	707 (49.3)	775 (45.6)	2 > 1 > 4 > 3 (1.04/3.59)
ACC4 (n)	**174 (20.6)**	**188 (22.3)**	144 (18.5)	160 (17.4)	2 > 1 > 4 > 3 (0.72/2.30)
DEC4 (n)	**277 (24.3)**	**304 (23.9)**	234 (20.7)	265 (21.5)	2 > 1 > 4 > 3 (0.56/3.12)

**Note:** 1, 2, 3 and 4 are the clusters. ES is effect size. TD is total distance, TD21 is distance covered at > 21 km · h^−1^, TD24 is distance covered at > 24 km · h^−1^, TD28 is distance covered at > 28 km · h^−1^, aLoad is acceleration load, ACC3 is number of accelerations at > 3 m · s^−2^, DEC3 is number of decelerations at < -3 m · s^−2^, ACC4 is number of accelerations at > 4 m · s^−2^, DEC4 is number of decelerations at < -4 m · s^−2^, and au is arbitrary unit.

**FIG. 1:**
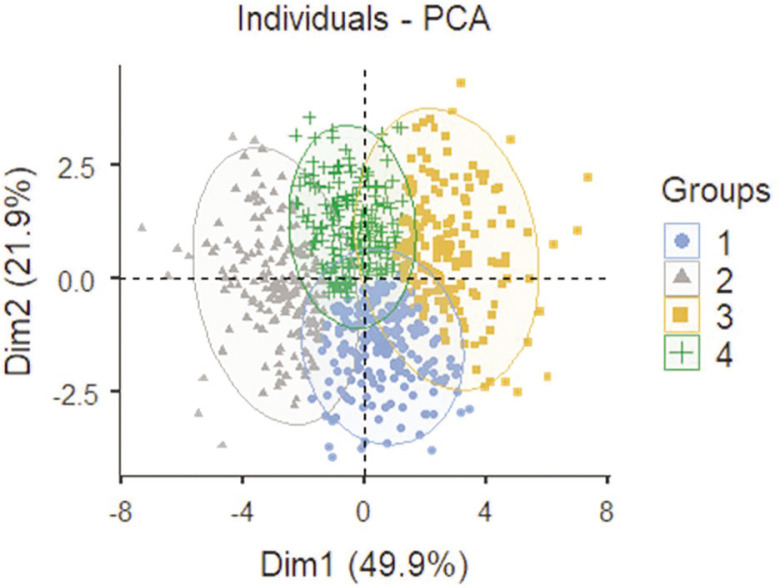
Distribution of the external match load data in the two first dimensions of the PCA analysis (PC1 and PC2), coloured by their belonging to one of the four clusters.

Figure 1 shows the distribution of external match load considering the first two PCs of the PCA analysis (PC1 and PC2) while colouring each point (team’s external match load data) according to their belonging to one of the four clusters.

As shown in Table 4, some teams have many matches belonging to a cluster, with significant differences in the distribution of their physical performance (χ^2^ = 257; df = 57; *p* < 0.001). Thus, the teams with the highest number of matches belonging to cluster 1, characterized by high value in volume (aLoad) and intensity (ACC/DEC variables), are Barcelona, Celta de Vigo, Mallorca, and Rayo Vallecano. In cluster 2, characterized by the highest values in all match load variables, only Athletic Club had a greater representation of physical performance in this cluster; that is, their matches are played almost always with high demands in volume, velocity and intensity. In cluster 3, characterized by low values for all variables, the teams with the most matches are Cádiz, Osasuna, Real Betis, Sevilla, Valencia and Villarreal. Finally, in cluster 4, characterized by high values in high-speed and sprint movements and moderate values in the rest of the variables, the teams were Atletico de Madrid, Granada, and Las Palmas. The teams that distributed their performances across two or more clusters were Alaves, Girona, and Real Sociedad (clusters 1 and 2), Almeria (clusters 1 and 4), Real Madrid (clusters 3 and 4), and Getafe (four clusters).

**TABLE 4:** Distribution of the number of official matches according to the physical demands of each team belonging to each cluster (1, 2, 3 and 4).

Teams	Clustering				Total

	1	2	3	4	
Alaves	**17**	**14**	1	6	38
Almería	**13**	7	6	**12**	38
Athletic Club	11	**17**	5	5	38
Atletico de Madrid	10	4	7	**17**	38
Barcelona	**15**	9	3	11	38
Celta de Vigo	**19**	10	4	5	38
Cádiz	6	1	**25**	6	38
Getafe	**10**	**9**	**10**	**9**	38
Girona	**11**	**12**	7	8	38
Granada	6	10	3	**19**	38
Las Palmas	7	6	7	**16**	36
Mallorca	**18**	6	10	3	37
Osasuna	11	2	**15**	10	38
Rayo Vallecano	**15**	10	3	10	38
Real Betis	7	0	**20**	11	38
Real Madrid	5	2	**14**	**17**	38
Real Sociedad	**13**	**12**	6	6	37
Sevilla	1	2	**28**	7	38
Valencia	12	2	**14**	10	38
Villarreal	7	2	**21**	8	38
Total	214	137	209	196	756

The analysis also showed that the distribution of matches within clusters had some correlation with the teams’ final rankings. As shown in Table 5 (χ^2^ = 11.9, df = 6 and *p* = 0.065), teams qualifying for European competitions had fewer matches in cluster 2 and were distributed more evenly across the other clusters. Specifically, teams qualifying for Europe had 27% of their matches in cluster 1 and 28% in cluster 4. Teams that maintained their league status had a higher percentage of matches in cluster 1 (31%) and cluster 3 (30%). Teams that faced relegation had a significant portion of their matches in cluster 4 (32%) and cluster 3 (30%).

**TABLE 5:** Distribution of the number of matches (n and %) according to the physical demands of each team ranking belonging to each cluster (1, 2, 3 and 4).

Team ranking		Clustering				Total

		1	2	3	4	
Europe	n	**72**	56	62	**75**	265
	%	**27**	21	23	**28**	

Maintain	n	**117**	63	**113**	84	377
	%	**31**	17	**30**	22	

Relegation	n	25	18	**34**	**37**	114
	% 22	16	**30**	**32**		

Total	n	**214**	137	**209**	196	756
	%	**28**	18	**28**	26	

Table 6 shows that teams’ physical performances tended to be played, for most of the matches, against opposing teams from the same cluster: teams in cluster 1 physical performances played 48% of their matches against cluster 1 opponent teams; teams in cluster 2 physical performances played 54% of their matches against cluster 2 opponent teams; and teams in clusters 3 and 4 physical performances faced cluster 3 and cluster 4 opponent teams in 56% and 53% of the matches, respectively (χ^2^ = 359.5, df = 9 and *p* < 0.001).

**TABLE 6:** Distribution home team physical performances (n and %) according to the rival physical performance belonging to each cluster (1, 2, 3 and 4).

Team clustering		Opposing team clustering				Total

		1	2	3	4	
1	n	**66**	31	5	35	137
	%	**48**	23	4	26	

2	n	31	**116**	48	19	214
	%	14	**54**	22	9	

3	n	5	48	**118**	38	209
	%	2	23	**56**	18	

4	n	35	19	38	**104**	196
	%	18	10	19	**53**	

**Total**	n	137	214	209	196	756
	%	18	28	28	26	

## DISCUSSION

This study aimed to describe the physical performance of teams during official matches using a multivariate approach and, additionally, to determine the association between teams’ physical performance, their opponents’ performance, and their final ranking in the competition. The results of the study made it possible to distribute the types of external load demands of each team in matches during a season into four profiles: high or low demand in all the variables analysed, low demand in high-speed range speed variables (distance at > 21, > 24 and > 28 km · h^−1^) and high demand in accelerations and decelerations (> 3 and > 4 m · s^−2^), and high demand in highspeed range variables and low demand in accelerations and decelerations. The PCA and clustering results provided a detailed understanding of the possible scenarios where teams competed, significant associations between teams, team ranking and team interaction.

The primary finding was the presence of three PCs with an eigenvalue greater than 1, which captured ~85% of the total external match load variance, with ~75% of the variance captured within the first two PCs (Table 2). The first transformed PC captures the most information (~54%), with each subsequent PC explaining a reduced amount of information. Within this PC1, several external match load variables contribute to changes in speed; both acceleration and deceleration actions (e.g., intensity) are included in this component. In the second of the components, which captured 21% of additional external match load information, the variables related to the actions performed in high-speed running and sprinting are essential (e.g., high intensity). These findings are similar to those found in previous studies [7, 22, 23], where the distance covered at high speed features prominently in the second of the PCs, complemented by more global load variables such as the PlayerLoad or total distance covered [7, 20]. In the third of the components, which captured 11% of additional external match load information, it seems that the variables related to the volume (total distance covered and acceleration load) are essential. These findings are significant because the most studied external match load variable in football, total distance covered, appears in this third component, providing more residual information about the teams’ activity during the competition. This is probably because these types of variables referring to volume do not have a direct relationship with success in competition (e.g., accumulating more points in competition, winning the match) [25, 26], and can perhaps be better explained as a consequence of team playing styles [5, 25] or contextual variables [15, 16, 35].

The current possibility of integrating different physical dimensions in team performance analysis [5, 16] allows for a better interpretation of physical performance in competition. It is known that teams show different physical responses according to the play style [24, 25]. It is also known [26] that situational variables influence the strategy teams adopt in the competition; therefore, the physical response will be altered [24]. This explains why teams exhibit variability in physical performance throughout the games during the season.

The multivariate analysis identified four types of physical performance of the teams during the competition. The physical performances of teams classified in cluster 1, with a moderate volume and high-intensity physical profile, suggest that these teams generally participated in matches with lower physical demands regarding high-speed running and sprint distance (TD21, TD24, and TD28). The physical performance of the teams classified in cluster 2, with the highest values in all physical variables, indicates that their matches involved high demand at all levels (volume, high speed and intensity), probably because they were ‘open’ matches with few stoppages. A recent study supports the idea that more effective playing time implies a higher physical demand for the teams [27], mainly because they have more time to accumulate more effort. The physical performance of the teams classified in cluster 3, with the lowest values in all physical variables, indicates a low physical demand, probably due to the matches being characterized by many interruptions of play (set pieces), where defensive aspects played a more significant role. Finally, the physical performance of the teams classified in cluster 4 was highly intense, probably with many box-to-box races. Teams with more direct play styles [24] or matches with little effective playing time [5] could be two reasons for this increase in the physical intensity demanded. In the information age, reducing the complexity of both the preparation process [8] and physical performance in competition [10] is becoming increasingly necessary to facilitate decision-making. This new approach, which groups variables into dimensions and profiles of physical performances in competition, allows coaches to easily classify match demands into one of these four groups, anticipating what can be expected from a team with a higher physical profile distribution in one of the groups. This, in turn, enables them to make informed decisions to prepare for the match. Additionally, this ranking may have implications for post-match recovery.

Scientific evidence supports the hypothesis that a better physical response does not correlate with success, understood as winning matches [25] or being ranked higher at the end of the competition [26]. However, the momentary score is a relevant factor for the physical response to be modified during the match [28], highlighting the need for more sensitive scoreline definitions in which to consider scoreline effects. As shown in Table 5, there were no significant differences in the final performance results regarding cluster distribution. However, teams that qualify for European competitions have fewer matches in cluster 2, and teams that maintain their league status have a slightly higher percentage of matches in cluster 1. This distribution could reveal different patterns of play. Probably for this reason, in recent years, attempts have been made to incorporate physical responses into the description of teams’ playing styles rather than associating them with success [21, 25, 29–32]. Furthermore, due to increasing competitive demands of each match and the succession of matches, it has become necessary to interpret these demands on a match-by-match basis.

It should be noted that, although some teams have a marked physical profile (probably because they are faithful to a model of playing), it is usual among LaLiga teams that there is a deployment of the physical response in all four clusters, e.g. that teams, throughout the championship, will have to respond to all the physical profiles in competition. This variability is due, above all, to the fact that, during the season, the situational variables of the match in dispute, e.g., place, score, weather [32, 33], as well as the contextual variables that arise, such as the dismissal of coaches [34], phase of the season [35], congested periods of competition [35], and prolonged injuries of essential players in the team [36], mean that teams must adapt their match strategies, necessitating an adaptive response to the different demands of the game and opponents [25]. This suggests that these teams need a versatile approach, adapting to match demands and conditions.

Regarding the interaction of teams’ physical performances, the findings of this study align with the conclusions drawn by Castellano et al. [16]. The study posits that the physical performance of football teams is highly interdependent, with one team’s physical output directly influencing that of its opponents. The clustering results further support this notion, demonstrating a significant correlation between the physical demands experienced by teams and their opponents during matches. This high association suggests that teams’ performances are not only reflective of their capabilities but also conditioned by their rivals’ playing styles and physical demands. This would justify the results obtained in the present study (see Table 6), where a team’s physical performance profile (cluster) coincides with that of its opponent in approximately 50% of the official matches. Then, both teams in their confrontation display a similar physical response (cluster 1 vs cluster 1, cluster 2 vs cluster 2, etc.). However, this higher probability does not exclude that there may be other match-ups with a different physical profile.

This research study has limitations. In the first place, although the sample was extensive, since all the performances of the teams in a complete season were involved, the performances were not differentiated according to contextual and situational variables such as time of the season, change of coach, injury of essential players, weather, expulsions, etc., which are known to affect the physical performance of the teams [26]. Considering all these particularities would have prevented us from gaining a global picture of the league’s teams. Secondly, the number and type of external match load variables chosen could have conditioned the analyses implemented; speed peaks, internal load variables or mechanical variables could have complemented the results described. Finally, incorporating other performance dimensions into the analysis, such as variables identifying play styles or the teams’ collective tactical behaviours, allowed a more complete and substantiated interpretation of the results. For the above reasons, the results should be interpreted cautiously, underscoring the need for more holistic approaches in analysing the results.

## CONCLUSIONS

In summary, the clustering analysis highlights significant variations in the physical demands experienced by different teams throughout the season. Most teams had a balanced spread across the clusters, with notable exceptions where specific teams consistently fell into clusters representing higher or lower physical demands. This comprehensive overview of how teams’ physical performances are distributed across clusters can inform training and match preparation strategies, helping teams optimize their performance based on the specific physical demands they encounter. Understanding these patterns can also aid injury prevention and recovery management by aligning physical preparation with the observed match load characteristics.
